# Editorial: Community series in mental-health-related stigma and discrimination: prevention, role, and management strategies, volume II

**DOI:** 10.3389/fpsyt.2024.1384836

**Published:** 2024-02-27

**Authors:** Renato de Filippis, Samer El Hayek, Mohammadreza Shalbafan

**Affiliations:** ^1^ Psychiatry Unit, Department of Health Sciences, University Magna Graecia of Catanzaro, Catanzaro, Italy; ^2^ Medical Department, Erada Center for Treatment and Rehabilitation in Dubai, Dubai, United Arab Emirates; ^3^ Mental Health Research Center, Psychosocial Health Research Institute, Department of Psychiatry, School of Medicine, Iran University of Medical Sciences, Tehran, Iran

**Keywords:** community psychiatry, internalized stigma, mental health, misinformation, public health, social stigma, stereotyping, stigmatization

Stigmatizing attitudes towards individuals experiencing mental illnesses, as well as their caregivers, mental health providers, psychotropic medications, mental health institutions, and stakeholders, persist as a prevalent public health concern with global widespread consequences (1). Undoubtedly, despite increased knowledge about mental health, societal misconceptions persevere, resulting in discrimination and the marginalization of those contending with mental health issues (2, 3). This matter is prominently observed in medicine, though, not exclusively, in individuals with mental disorders and its prevalence has intensified in the aftermath of the COVID-19 pandemic (4–6).

In this second volume of the Community Series entitled *Mental-Health-Related Stigma and Discrimination: Prevention, Role, and Management Strategies*, we present 22 new articles exploring various facets of mental-health-related stigma and discrimination, offering diverse perspectives from different countries (7). Through this editorial, we endeavor to encapsulate the key points from these articles and encourage the audience to delve into the comprehensive insights provided in this Research Topic.

Among the works included in this Research Topic, five of them assessed the existing relationship between stigma, mental health care providers, and stakeholders. The qualitative study by Hajebi et al. addresses the impact of stigma on mental health patients in Iran, leading to reluctance in seeking help and discontinuing treatment due to fear and embarrassment. Authors involved purposive sampling and utilized focus group interviews with mental health stakeholders. Thirteen participants, including psychologists, psychiatrists, managers, patients, and a family member discussed challenges, solutions, and successes related to stigma management in Iran. The findings emphasize the importance of raising awareness and providing training to diverse groups, including patients, families, therapists, leaders, policymakers, the public, and the media to change existing stereotypes and reduce stigmas. In another qualitative study lead by Badrfam et al., they examine the stigma experienced by frontline healthcare workers (HCWs) during the initial COVID-19 wave in Iran. The study identified four themes, eight categories, and 33 sub-categories encompassing extrinsic elements like “creating blame and shame” and “discrimination,” intrinsic elements like “the desire to be avoided,” “feeling depressed and frustrated,” and “feeling anxious and scared,” as well as perplexity and stigma removal requirements. Factors contributing to stigma among HCWs included low public awareness of COVID-19, insufficient public care, limited protective equipment, inadequate facilities, lack of appreciation, and a deficit in mental health support. Chuen Yu et al. conducted a transnational study, utilizing the Health Stigma and Discrimination framework (HSDF), employing semi-structured informant interviews with non-probability sampling, focusing on public perceptions and reactions to the pandemic in a multicultural context, with specific attention to findings from Singapore in Asia. Twenty-nine participants aged 23 to 80 years were interviewed, and the thematic analysis of coded interviews revealed five major themes: perception and experiences of stigma among respondents, drivers of stigma and misinformation, facilitators for prevention and reduction, and ageist attitudes toward older adults. Through the HSDF, the study provides an exploratory account of COVID-19-induced stigma in an Asian context, highlighting the importance of trust and effective communication in mitigating stigma during public health crises. Still, Huang et al. explored the attitudes and intentions of Chinese HCWs towards seeking professional psychological help amid the COVID-19 pandemic. Authors approached 1,224 participants from 12 hospitals in Hunan province, China, administrating the Attitudes Toward Seeking Professional Psychological Help Scale-Short Form (ATSPPH-SF) and the General Help-Seeking Questionnaire (GHSQ). Results from 1,208 HCWs revealed generally negative attitudes and low intentions regarding seeking professional psychological help during the pandemic. Additionally, psychological learning experience and social support positively influenced intentions to seek professional psychological help, whereas divorced marital status and self-stigma had negative effects. Finally, Kamalzadeh et al. wrote an opinion piece about perspectives from Early Career Psychiatrists (ECPs) Section members of the World Psychiatric Association (WPA), with the aim to explore the influence of stigma on the location and configuration of mental health establishments. The inquiry also investigated its effects on the professional identities and job satisfaction levels of psychiatrists across ten different national contexts, including India, Indonesia, Iran, Italy, Lebanon, Malaysia, Nigeria, Thailand, Tunisia, and the United Kingdom. Recommendations for enhancing the quality and accessibility of mental health care were also provided.

Two articles addressed psychosocial issues in light of stigma in mental health. In the first one Jain et al. ran a review examining the impact of stigma and psycho-socio-cultural challenges on the control of the COVID-19 pandemic. Their findings highlighted the influence of various psychosocial, socio-economic, and ethno-cultural factors on the transmission and control of COVID-19: indeed, stigma and related psychosocial challenges, including anxiety, fear, and stigma-driven social isolation have significantly contributed to mental health issues. Then, Helmert et al. explored variations in the desire for social distancing from individuals with mental illness by analyzing social and spatial information. The study found that stigma levels varied among city districts, and a higher desire for social distance was associated with spatial differences, increased pessimism, heightened shame about mental illness, lower social support, lower socio-economic status, and older age. The results highlight the importance of the geographical context in understanding and addressing mental health stigma.

Two articles that assessed the role of art in combating stigma were included. In the first one, Moeenrad et al. described the experience of the “Art and Psyche Festival”, based on the application of protest, education, and/or interpersonal contact with someone affected by mental health issues. Authors concluded that the festivals, aimed at gathering social attention and support, did not formally evaluate the anti-stigma impact. Nevertheless, the organizers hypothesized that incorporating research methods into a third festival can further support the belief in the facilitating role of art in destigmatizing psychiatric disorders. Moreover, El Halabi et al. led a group of 12 ECPs from different countries and cultures collecting data and professional experiences about the role, function, and impact of art in counteracting stigma in mental health in their respective countries. The authors concluded that art can play a decisive role in improving the conditions of treatment and rehabilitation in psychiatry, but a lot still needs to be done, and such potential remains to be enormously enhanced and implemented, almost everywhere in the world.

The stigma associated with substance use disorder (SUD) was explored in depth by four different articles. Sapag et al. proposed a study protocol with the aim to assess the effectiveness of an anti-stigma intervention in reducing stigmatizing attitudes and behaviors among mental health providers toward individuals with mental illness and/or SUD in Chile. This research aimed to advance mental health and stigma research in Chile, contributing to improved access and quality of care for individuals with SUD. Evaluating the intervention’s impact and implementation will provide insights for scaling it up to other “Centros de Salud Familiar” across Chile. Henderson et al. published an original research article including 133 individuals under treatment, wherein they assessed the influence of substance use/misuse risk factors and looked at perceived societal stigma and self-stigma. Their findings provide additional insights into the intricate relationship between culture and the individual, emphasizing the role of cultural distance in shaping self-stigma among those under treatment for substance use issues. In another opinion piece, El Hayek et al. proposed a multinational perspective and call for action on stigma toward SUD. Authors highlighted the urgent need for recognizing the significant challenges posed by stigma and reevaluating the language used in discussions about addiction. Additionally, the decriminalization of drug use was emphasized as a crucial step, not only in diminishing stigma but also in reallocating resources toward prevention and treatment, advocating for an approach that prioritizes healing over punishment. Finally, Cunningham et al. aimed to uncover instances of discrimination by clinicians, exploring the role of clinician beliefs and assumptions in the provision of physical health services for individuals with SUD. The study surveyed 253 patients who had accessed physical healthcare services about their experiences. The findings underscored the impact of discrimination based on SUD on the quality of care. The study emphasized the need for health systems and clinicians to prioritize quality improvement processes that ensure equitable access to and delivery of physical healthcare for individuals with SUD.

Focusing on the influence of COVID-19, three studies dedicated more attention to the role of the pandemic. Azman et al. tried to assess posttraumatic growth and its associations with stigma, psychological complications, and sociodemographic factors among COVID-19 patients six months post-hospitalization. Factors predicting posttraumatic growth included a higher level of perceived stigma, Malay ethnicity, retired status, and a history of medical illness. In a nutshell, the study suggested that experiencing stigma contributed to posttraumatic growth in COVID-19 patients, alongside sociodemographic and psychosocial factors. Shah et al. designed a cross-sectional study in Nepal with the goal to assess various aspects related to 395 individuals admitted for COVID-19 or suspected cases, including sociodemographic details, clinical information, COVID-19-related knowledge, perception, internalized stigma, and symptoms of depression and anxiety. Key findings included that 23.3% of patients had anxiety symptoms, 32.9% had depressive symptoms, and 20.3% experienced high COVID-19-related internalized stigma. The third study was an online survey by Hu et al. conducted in China, and covering all provinces, autonomous regions, and municipalities. It aimed to investigate how three dimensions—individual resilience perception, community resilience perception, and government trust perception—mitigate anxiety during COVID-19. Additionally, there was a positive correlation between community resilience perception, government trust, and individual psychological resilience. Government trust perception was found to enhance psychological resilience, leading to a reduction in anxiety. In summary, individual psychological resilience, community resilience perception, and government trust perception played crucial roles in mitigating anxiety during the COVID-19 pandemic, providing valuable insights for understanding mental well-being in challenging times.

Two papers investigated the relationship between mental health-related stigma and students. In one study, Porfyri et al. explored the views of Greek medical students toward mental illness and patients. They conducted a cross-sectional study involving 324 undergraduate students from the Aristotle University of Thessaloniki. While the findings align with previous studies, they suggested an improvement compared to earlier research among Greek student and healthcare populations. The study emphasized the need for ongoing vigilance, educational interventions, and social initiatives to empower current and future healthcare professionals to fulfill their roles effectively. In the study by Zavorotnyy et al., the authors investigated the impact of a psychiatric clerkship on stigmatizing attitudes toward mental disorders among 256 third- and fourth-year medical students in pre- and post-clerkship surveys. The study suggested that a psychiatric clerkship involving direct patient interaction can effectively decrease stigma. The findings support the incorporation of such components in medical education to combat stigma, potentially improving outcomes for individuals with severe mental disorders.

Two publications dealt with psychometric assessments in mental health-related stigma. In the first one, Peng et al. investigated expressed emotion, referring to family members’ attitudes and emotional behaviors toward mentally ill relatives. This research successfully translated, adapted, and assessed the psychometric properties of a Chinese version of the Family Questionnaire. The questionnaire demonstrated a consistent two-factor structure (emotional over involvement and criticism), with reliability and validity confirmed through analyses of internal consistency, factor structure, and concurrent validity. In the second study, the research group led by Lu et al. focused on translating the Dementia Public Stigma Scale (DPSS) into standard written Chinese, developing a person-centered translation method, and proposing a tripartite assessment construct for translation quality evaluation. Authors were able to develop a method and an assessment construct for person-centered translation of dementia public stigma scales.

Pokharel et al. were able to collect ECPs’ perspectives about mental illness stigma among perinatal women in low- and middle-income countries (LMICs). In this paper authors focused on the need for stigma reduction initiatives specifically targeting perinatal mental disorders in LMICs. They concluded that these programs should integrate effective intervention components, including educational methods such as dispelling myths and increasing knowledge. The implementation of these evidence-based interventions, designed to reduce stigma and discrimination, holds the potential to enhance help-seeking behavior and improve access to appropriate mental health care in LMICs.

Finally, through a series of individual semi-structured interviews (n = 27), Chen et al. aimed to comprehensively examine the impact of the anti-Asian racism within a Chinese community in the greater Boston area. Participants advocated for increased education, community and governmental support, and enhanced allyship among communities of color. These findings offer a cultural context for understanding the trauma experienced by this population and can guide future initiatives aimed at addressing the diverse array of reported health effects.

In conclusion, the articles collected in this editorial focus on the need to develop a comprehensive strategy to overcome mental health-related stigma, encompassing public awareness initiatives, educational efforts, and the use of destigmatizing language. The future of psychiatry should concentrate on creating an atmosphere of empathy, understanding, and open dialogue, empowering individuals to seek help without fear of judgment. Collaborative endeavors involving mental health professionals, policymakers, and communities are vital to dismantle mental health-related stigma, paving the way for enhanced mental health outcomes and a more compassionate society (8–10).

## Author contributions

RF: Writing – original draft. SE: Writing – review & editing. MS: Writing – review & editing.
